# Standalone plyometric training in basketball players: a meta-analytic comparison of countermovement jump, squat jump, and sprint performance enhancements

**DOI:** 10.3389/fphys.2026.1747487

**Published:** 2026-03-18

**Authors:** Jinqi Liu, Shiwei Chen, Fuyou Yu, Qingbo Meng

**Affiliations:** 1 School of Physical Education and Health, Qufu Normal University, Qufu, China; 2 School of Health Sciences, Universiti Sains Malaysia, Kelantan, Malaysia

**Keywords:** basketball players, countermovement jump, plyometric training, sprint performance, squat jump

## Abstract

**Introduction:**

While several meta-analyses have quantitatively examined plyometric training effects, many combined it with other modalities (e.g., strength training) or did not focus exclusively on basketball players. This systematic review and meta-analysis therefore aimed to quantitatively evaluate the independent effect of plyometric training on lower-body explosive power and sprint performance specifically in basketball athletes, focusing on countermovement jump (CMJ), squat jump (SJ), and 20-meter sprint outcomes.

**Methods:**

A comprehensive search was conducted across PubMed, Web of Science, Embase, Cochrane Library, and SportDiscus up to 24 September 2025. Randomized controlled trials and quasi-experimental studies comparing plyometric training with control conditions in basketball players were included. A random-effects model was used to calculate pooled Hedges’ g with 95% confidence intervals. Heterogeneity and publication bias were assessed using I^2^ statistics and funnel plots with Egger’s test.

**Results:**

Seventeen studies involving 444 participants were included. The meta-analysis revealed significant improvements in countermovement jump (g = 0.77, 95% CI 0.42–1.11) and squat jump (g = 0.86, 95% CI 0.52–1.20) following plyometric training, with minimal heterogeneity for squat jump (I^2^ = 0.5%) but considerable heterogeneity for countermovement jump (I^2^ = 67.6%). A small but significant effect was found for 20-meter sprint performance (g = −0.39, 95% CI −0.72 to −0.05), with no heterogeneity (I^2^ = 0%). Subgroup analysis based on training duration showed no significant difference in countermovement jump outcomes.

**Conclusion:**

Standalone plyometric training is effective for enhancing vertical jump performance in basketball players, particularly squat jump, but has limited transfer to 20-meter sprint performance. Future research should focus on standardized intervention designs and consider moderating factors such as training volume and athlete characteristics.

**Systematic Review Registration:**

https://www.crd.york.ac.uk/prospero/display_record.php?ID=CRD420251179252, identifier CRD420251179252.

## Introduction

Basketball strength and conditioning programs place significant emphasis on developing power and speed capabilities, which are essential for executing fundamental game actions such as jumping, linear sprints, accelerations, decelerations, and directional changes performed repeatedly in both offensive and defensive scenarios (Stojanović et al., 2018; Scanlan et al., 2011). Among these physical attributes, lower-body explosive power represents a crucial performance determinant, directly impacting a player’s capacity for rebound challenges, shot blocking, and rapid acceleration in open-court situations (Šarabon et al., 2020). Given this association with on-court performance, the countermovement jump (CMJ, with pre-stretch buffering), squat jump (SJ, without pre-stretch, focusing on pure concentric contraction), and short-distance sprints serve as classic tests of lower-body power and acceleration. Specifically, SJ effectively evaluates the concentric-dominant explosive strength associated with basketball-specific movements such as immediate vertical takeoff during jump shots without pre-squat, or sudden rebound grabs in crowded penalty areas, as established in the basketball literature (Castagna et al., 2013; Santos and Janeira, 2011).”

To enhance these athletic qualities, plyometric training has become fundamental to basketball conditioning regimens (Ramirez-Campillo et al., 2018). Grounded in the exploitation of the stretch-shortening cycle (SSC), plyometric exercises comprising various jumps and hops are designed to produce rapid, powerful movements (Markovic and Mikulic, 2010). The underlying physiological mechanism involves a pre-stretch of the muscle-tendon unit that stores elastic energy, thereby facilitating enhanced subsequent concentric contraction (Komi, 2000). This neuromuscular process induces adaptations that improve both neuromuscular efficiency and rate of force development (Taube et al., 2012).

A substantial body of primary research has examined the effects of plyometric training on basketball performance. For instance, numerous studies—including those by Castagna et al. (2013), Ramirez-Campillo et al. (2018), and Šarabon et al. (2020)—have specifically investigated its impact on key performance metrics. These include improvements in vertical jump height—encompassing both countermovement jump and squat jump performance—in junior players following a structured program (Shibli et al., 2018). Significant enhancements in squat jump capacity have also been observed in adolescent male athletes (Santos and Janeira, 2011). For young female athletes, plyometric training has been shown to improve sprint acceleration and agility (Attene et al., 2015). Additionally, positive changes in change-of-direction ability have been reported following complex training interventions that incorporate plyometrics (Cavaco et al., 2014). Collectively, this primary literature forms a robust evidence base exploring the efficacy of plyometric exercises for basketball-specific athletic development. However, existing syntheses are limited in several key aspects. First, they often confound the unique contribution of plyometrics by combining it with other training modalities (e.g., strength training) (Bouteraa et al., 2020). Second, many rely on qualitative summaries rather than comprehensive meta-analyses, thus failing to provide pooled effect estimates (Moran et al., 2019). Building upon this foundation, this systematic review and meta-analysis quantitatively synthesizes evidence for the effects of standalone plyometric training on countermovement jump, squat jump, and 20-meter sprint performance in basketball players, thereby establishing an evidence-based foundation for optimizing basketball-specific plyometric training prescriptions.

## Methods

This review was performed in accordance with the PRISMA statement (Page et al., 2021), and the protocol was prospectively registered in the PROSPERO database (CRD420251179252).

### Search strategy and selection

A systematic literature search was performed utilizing multiple electronic databases, specifically PubMed, Web of Science, Embase, the Cochrane Library, and EBSCOhost-SportDiscus. The search covered all publications available from the inception of each database until 24 September 2025. Key concepts incorporated into the search strategy consisted of terms associated with plyometric training (such as “plyometric,” “jump training,” and “explosive training”), basketball athletes (including “basketball,” “basketball player,” and “athlete*”), as well as relevant performance indicators like countermovement jump, squat jump, and sprint performance. Boolean operators (“AND” and “OR”) were used to link these concepts, and controlled vocabulary terms (e.g., MeSH in PubMed) were applied where appropriate. The complete search strategy is available in Supplementary Material 1.

After eliminating duplicate records, the titles and abstracts of the remaining citations underwent a preliminary assessment for relevance by two independent reviewers (JL and SC). The full texts of studies deemed potentially eligible were then retrieved and evaluated based on the predefined inclusion criteria. Any discrepancies between the reviewers during the selection process were resolved through discussion with FY, with any persisting disagreements adjudicated by QM.

### Eligibility criteria

Study eligibility was determined based on the following predefined criteria: (a) the study population consisted of basketball players; (b) the intervention was a structured plyometric training program; (c) a control group was present, receiving either no specific plyometric training or continuing their regular training routine; and (d) the study reported quantitative results for at least one of the following performance outcomes: countermovement jump height, squat jump height, or 20-meter sprint time. Acceptable study designs were restricted to randomized controlled trials or quasi-experimental studies that included both an intervention and a control condition. Furthermore, included studies were required to provide adequate post-intervention data, specifically means and standard deviations, to permit the computation of effect sizes.

Studies were excluded from the analysis for any of these reasons: the participants were not identified as basketball players; the study design lacked a control or comparison group; the implemented intervention did not primarily consist of plyometric training; the report omitted the relevant performance outcomes (countermovement jump, squat jump, or 20-m sprint); or the available statistical data were insufficient for calculating effect sizes.

### Data extraction

A standardized data edxtraction procedure was implemented to ensure systematic collection of relevant information from all eligible studies. The extraction process was conducted independently by three reviewers (JL, SC, and FY) using a pre-designed data collection form. The following data fields were systematically recorded: first author’s name, year of publication, country where the study was conducted, research design, participant demographics (including total sample size, average age, and sex composition), intervention parameters (encompassing training modality, session frequency, program duration, intensity indicators [e.g., jump height, ground contact time, loading], specific plyometric exercises [e.g., depth jumps, box jumps, bounding]), and potential moderating variables (including age group, sex, basketball competition level, training volume, and intervention length).

For quantitative synthesis, baseline and post-intervention data were extracted for both experimental and control groups, including mean values and corresponding standard deviations for all primary outcomes. When studies directly reported change scores (calculated as post-intervention minus baseline values), these were utilized for analysis. In cases where change scores were not provided but adequate raw data were available, they were computed manually.

Consistency among data extractors was ensured through iterative cross-checking procedures, and any persisting discrepancies were resolved through consultation with a senior researcher (Q.M.) for final determination.

### Risk of bias

The methodological quality and risk of bias of the included studies were evaluated using the ROBINS-I tool. This instrument systematically assesses potential biases across seven key domains: confounding factors, participant selection procedures, intervention classification, deviations from intended interventions, handling of missing data, outcome measurement methods, and selection of reported results. Each domain was categorized as demonstrating low risk (study comparable to a well-performed randomized trial), moderate risk (study appears sound but cannot be considered comparable to a randomized trial), serious risk (study has important problems), or critical risk (study is too problematic to provide useful evidence), following the ROBINS-I guidance (Sterne et al., 2016). Two reviewers (JL and SC) independently performed these evaluations. Initial disagreements between assessors were resolved through discussion with a third reviewer (QM), who also provided definitive judgment for any remaining unresolved discrepancies.

### Publication bias and sensitivity analysis

Potential publication bias was evaluated through a combination of visual inspection of funnel plots and quantitative statistical testing using Egger’s regression method (Egger et al., 1997). Funnel plot asymmetry was interpreted as non-significant when the associated p-value exceeded 0.05. To test the stability and reliability of the meta-analytic findings, sensitivity analyses were implemented using a two-stage approach: initially by removing studies judged to have a high risk of bias, and subsequently by systematically excluding each individual study in turn according to the leave-one-out procedure (Viechtbauer and Cheung, 2010). All analytical methods were aligned with the standards and guidance outlined in the Cochrane Handbook (Higgins et al., 2024).

### Statistical analysis

Subgroup analyses were conducted based on training duration (≤6 weeks vs. >6 weeks), age group (adolescents ≤18 years vs. adults >18 years), and sex (male vs. female) to explore potential sources of heterogeneity. When sufficient studies were available, meta-regression was performed to examine the continuous effect of age on outcomes. A series of meta-analyses were conducted to quantify the effects of plyometric training on primary basketball performance metrics, utilizing pre-post differences (calculated as post-intervention minus pre-intervention values) from both intervention and control groups. Treatment effects were expressed as Hedges’ g with corresponding 95% confidence intervals to account for potential small-sample bias. All analyses employed a random-effects model to accommodate expected clinical and methodological variations among the included studies. Between-study variance was estimated using the restricted maximum likelihood method, and the Hartung-Knapp adjustment was applied for confidence interval calculation to enhance the reliability of the results.

Heterogeneity among studies was evaluated using the I^2^ statistic, with values of 25%, 50%, and 75% representing low, moderate, and substantial heterogeneity, respectively. For outcomes demonstrating considerable heterogeneity (I^2^ > 50%), subgroup analyses based on training duration were performed to explore potential sources of variation. All statistical computations were carried out using R statistical software (version 4.4.0) with the metafor package, applying a two-tailed significance threshold of p < 0.05.

## Results

### Study selection and basic characteristics

**FIGURE 1:**
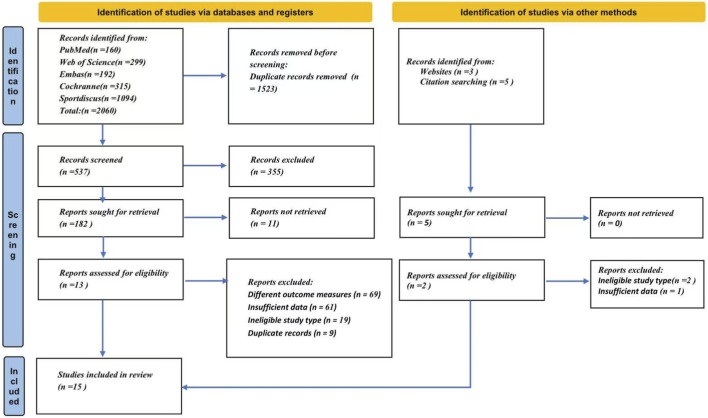
PRISMA 2020 flow diagram showing the process of study identification, screening, eligibility assessment, and inclusion.

The literature search conducted on 24 September 2025, yielded 2,060 records. Following a preliminary screening, 182 articles were shortlisted for further evaluation. After a full-text review of these studies, 15 fulfilled the eligibility criteria and were incorporated into the meta-analysis. de Villarreal et al., 2021; Pinheiro Paes et al., 2022; Haghighi et al., 2024; Aksović et al., 2019; Izquierdo Velasco, 2022; Sharma and Multani, 2012; Santos and Janeira, 2011; Khlifa et al., 2010a; Vescovi et al., 2008; Bavli, 2012; Attene et al., 2015; Meszler and Váczi (2019); Cherni et al., 2021; Santos and Janeira, 2008; Zribi et al., 2014. A detailed flowchart of the study selection process is shown in Figure 1.

The final sample of 15 studies comprised a total of 623 participants. Each study adopted a design that compared post-intervention outcomes between an exercise group and a non-intervention control group. The primary endpoints for analysis were performance in the countermovement,squat jump and the 20-meter sprint. The intervention duration varied across the included trials. A summary of the key study characteristics is provided in Table 1.

**TABLE 1:** Characteristics of included literature.

Author	Country	N (total sample)	Mesure	Training weeks	% Female	Session frequency (per week)	Age (years)	Key plyometric exercises
de Villarreal et al. (2021)	Spain	20	20 m sprint(S) countermovement jump (cm)	7 weeks	0%	2	13.57 ± 1.39	Depth jump, box jump
Pinheiro Paes et al. (2022)	Portugal	13	20 m sprint(S)	8 weeks	0%	2	15.83 ± 0.75	Countermovement jump, horizontal jump
Haghighi et al. (2024)	Turkey	16	20 m sprint(S)	6 weeks	100%	2	14.6 ± 1.5	Depth jumps, hurdle jumps
Aksović (2019)	Serbia	33	20 m sprint(S)	10 weeks	0%	2	15.0–16.0	Drop jump Counter movement jump
Izquierdo Velasco (2022)	Spain	32	20 m sprint(S)	8 weeks	0%	3	16.65 ± 1.24	Drop jump Box jump
Sharma and Multani (2012)	Indian	40	Countermovement jump (cm)	4 weeks	0%	3	12.0–20.0	Depth jump, box jump
Santos and Janeira (2011)	Portugal	24	Countermovement jump (cm) Squat jump (cm)	10 weeks	0%	2	14.5 ± 0.4	Depth jump, Medicine ball throw
Khlifa et al. (2010b)	Tunis	18	Countermovement jump (cm) Squat jump (cm)	10 weeks	0%	1–3weeks,2; 4–10weeks,3	23.57 ± 0.34	Drop jump, Vertical jumping
Vescovi et al. (2008)	American	20	Countermovement jump (cm)	6 weeks	100%	3	20.3 ± 1.2	Wall jumps, tuck jumps
Bavli (2012)	Turkey	24	Countermovement jump (cm)	6 weeks	0%	2	22.1 ± 2.4	Split squat jump, squat jump
Attene (2015)	Italy	36	Countermovement jump (cm) Squat jump (cm)	6 weeks	0%	2	14.83 ± 0.92	Drop jump from 40 cm box, front obstacle jumps with knees bending
Meszler and Váczi (2019)	Hungary	18	Countermovement jump (cm)	7 weeks	0%	4–5	15.8 ± 1.2	Double-leg hurdle jump, single-leg lateral cone jump
Cherni et al. (2021)	Tunisia	27	Countermovement jump (cm) Squat jump (cm) 20 m sprint(S)	8 weeks	100%	2	20.9 ± 2.4	Drop jumps, hurdle jumps
Santos and Janeira 2008	Portugal	25	Countermovement jump (cm) Squat jump (cm)	10 weeks	0%	2	14.7 ± 0.5	Depth jump, lateral box jump
Zribi et al. (2014)	Tunisia	51	Countermovement jump (cm) Squat jump (cm)	9 weeks	0%	2	12.1 ± 0.6	Box jump, hurdle jump

### Risk of bias and publication bias

**FIGURE 2:**
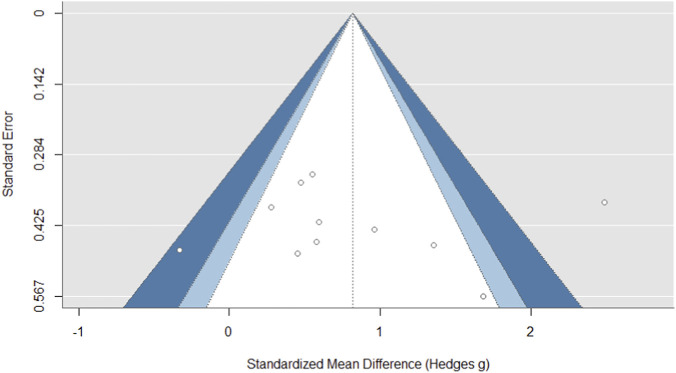
Risk of bias assessment of the included studies using the ROBINS-I tool.

The overall risk of bias for the included studies, as assessed by the ROBINS-I tool, was predominantly low to moderate (Figure 2). Low risk was frequently observed in the domains of participant selection (D2), missing data (D5), and selection of the reported result (D7). In contrast, biases were more common in the classification of interventions (D1), deviations from intended interventions (D3), and measurement of outcomes (D6), primarily attributable to a lack of blinding and non-standardized training protocols. No study was rated as having a serious risk of bias. Therefore, the methodological quality of the evidence base is considered acceptable, and the potential influence of bias on the overall results is likely limited.

### Sensitivity analysis

**FIGURE 3:**
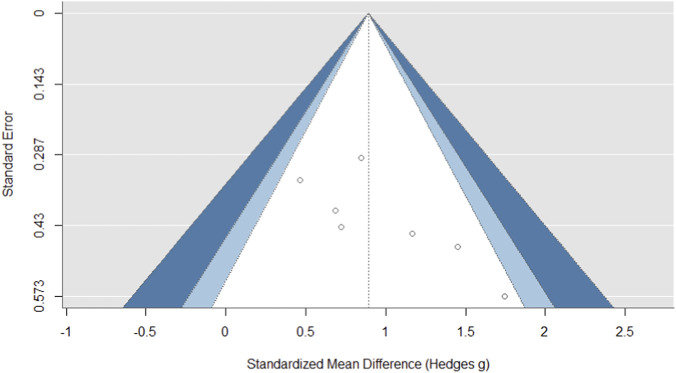
The funnel plot for the countermovement jump outcome showed evidence of publication bias.

For the countermovement jump outcome, the funnel plot exhibited a degree of asymmetry, characterized by an aggregation of smaller, less precise studies favoring higher effect sizes on the right, alongside a relative absence of studies with null or negative findings on the left (Figure 3). This visual suggestion of publication bias, however, lacked statistical corroboration, as Egger’s regression test was non-significant (z = 0.263, p = 0.792). Consequently, while the pooled countermovement jump estimate should be interpreted with caution due to the observed asymmetry, the absence of definitive statistical evidence for small-study effects must also be acknowledged.

**FIGURE 4:**
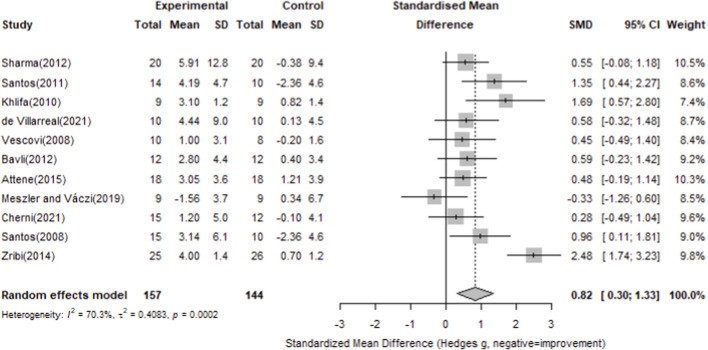
The funnel plot for the squat jump outcome showed evidence of publication bias.

For the squat jump outcome, the funnel plot demonstrated a certain degree of asymmetry, with a cluster of smaller, less precise studies showing more positive effect sizes on the right side and a relative lack of studies with null or negative findings on the left (Figure 4). This visual pattern indicating potential publication bias was not statistically confirmed, as Egger’s regression test did not reach formal significance (z = 1.766, p = 0.077). Therefore, while the observed asymmetry suggests that the pooled squat jump estimate should be interpreted with some caution, the lack of definitive statistical evidence for small-study effects must also be taken into account.

**FIGURE 5:**
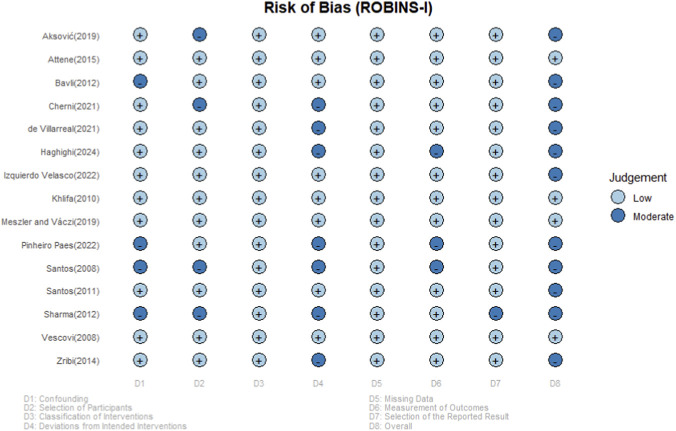
The funnel plot for the 20 m sprint outcome showed evidence of publication bias.

Statistical assessment of publication bias for the 20 m sprint outcome revealed no substantial evidence. The funnel plot demonstrated symmetry (Figure 5), and Egger’s regression test yielded a non-significant result (z = 0.014, p = 0.989), which does not support the presence of small-study effects. The even dispersion of effect estimates across the plot suggests a balanced representation of studies, regardless of their reported outcome magnitude. It can be concluded that publication bias is unlikely to have significantly influenced the pooled effect size, though the potential impact of other sources of methodological heterogeneity should be considered.

### Main effect results

#### Countermovement jump

**FIGURE 6:**
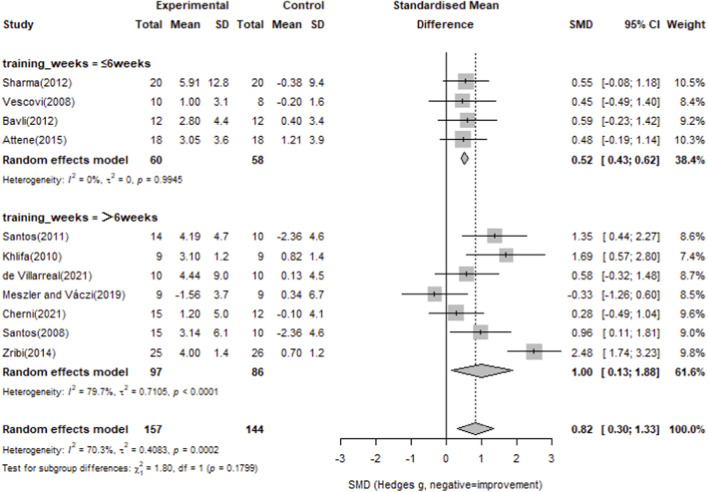
Forest plot of the effect of plyometric training on countermovement jump compared with controls (random-effects model).

For countermovement jump, twelve studies with 157 participants in the intervention groups and 144 in the control groups were included. The pooled analysis showed a significant overall effect of plyometric training compared with controls (Standardized Mean Difference = 0.82, 95% CI 0.30–1.33), with considerable heterogeneity (I^2^ = 70.3%, p = 0.0002). Most individual studies demonstrated positive effects on countermovement jump performance, as detailed in Figure 6.

**FIGURE 7:**
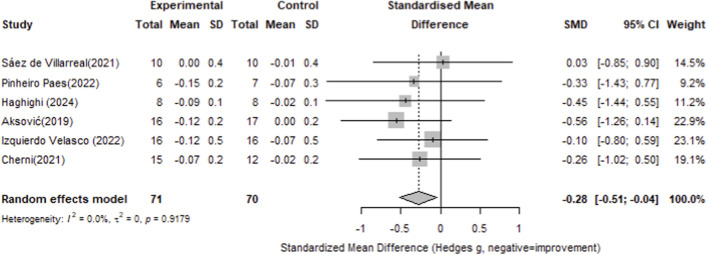
Subgroup analysis of countermovement jump by training weeks (≤6 weeks vs. > 6 weeks).

To compare the impact of training duration, a subgroup analysis was performed based on training weeks (≤6 vs. >6 weeks). The ≤6 weeks subgroup showed a significant effect (Hedges’ g = 0.52, 95% CI 0.43–0.62), and the >6 weeks subgroup also exhibited a significant effect (Hedges’ g = 1.00, 95% CI 0.13–1.38), with detailed results in Figure 7.

**FIGURE 8:**
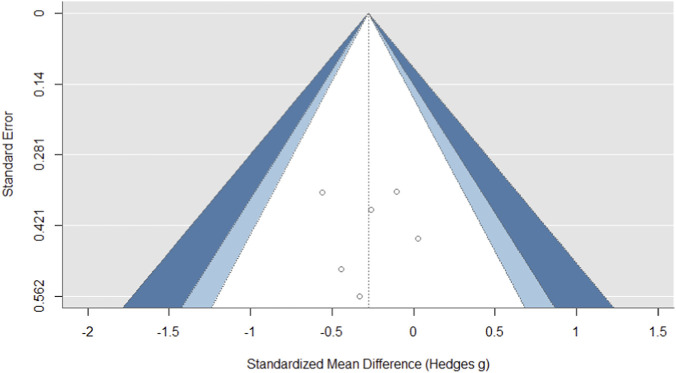
Forest plot of the effect of plyometric training on squat jump compared with controls (random-effects model).

To explore the potential influence of age, participants were divided into <18 and ≥18-year-old groups. The younger group showed a significant effect relative to controls (Hedges’ g = 0.93, 95% CI 0.07–1.79), while the older group did not (Hedges’ g = 0.05, 95% CI -0.25–1.55), as presented in Figure 8.

**FIGURE 9:**
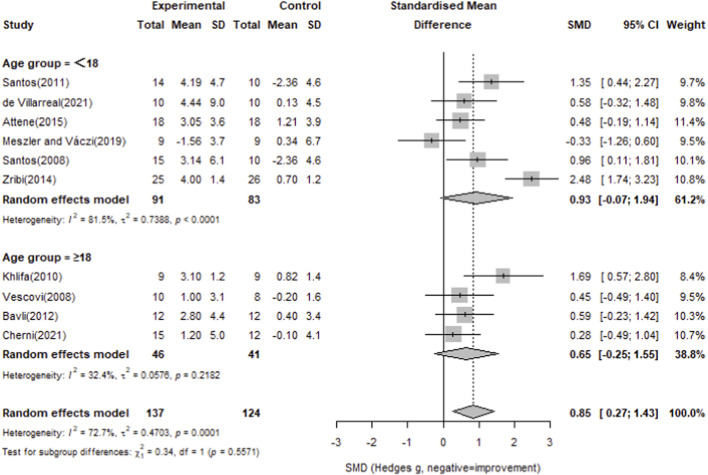
Forest plot of the effect of plyometric training on 20 m sprint compared with controls (random-effects model).

To examine gender differences, a subgroup analysis by gender (male vs. female) was conducted. The male subgroup demonstrated a significant effect (Hedges’ g = 1.16, 95% CI 0.47–1.84), whereas the female subgroup did not (Hedges’ g = 0.27, 95% CI -0.28–0.81); see Figure 9 for details.

#### Squat jump

**FIGURE 10:**
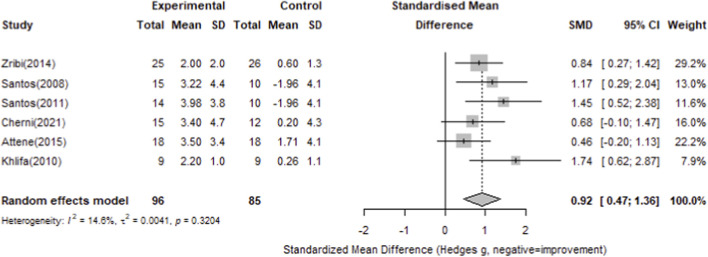
Forest plot of the effect of plyometric training on squat jump performance in basketball players (random-effects model).

For squat jump performance, seven studies with 108 participants in the intervention groups and 96 in the control groups were included. The pooled analysis showed a significant overall effect of plyometric training compared with controls (Standardized Mean Difference = 0.86, 95% CI 0.52–1.20), with minimal heterogeneity (I^2^ = 0.5%, p = 0.4185). All individual studies demonstrated positive effects on squat jump performance, as detailed in Figure 10.

#### 20-Meter sprint

**FIGURE 11:**
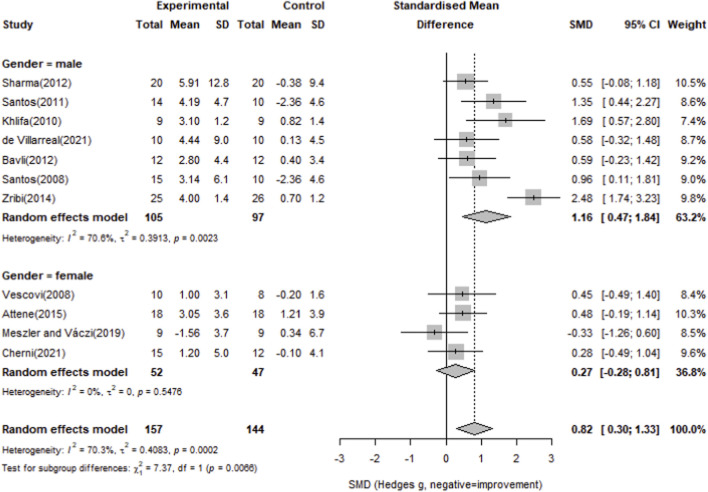
Subgroup analysis of the effect of plyometric training on countermovement jump performance by gender (male vs. female; random-effects model).

For 20-meter sprint performance, six studies with 71 participants in the intervention groups and 70 in the control groups were included. The pooled analysis showed a small but significant overall effect of plyometric training compared with controls (Standardized Mean Difference = −0.28, 95% CI -0.51 to −0.04), with no observed heterogeneity (I^2^ = 0.0%, p = 0.879). with the specific data detailed in Figure 11.

## Discussion

This systematic review and meta-analysis synthesized existing evidence to evaluate the effects of stand-alone plyometric training on the physical performance of basketball players, specifically focusing on the countermovement jump, squat jump, and 20-meter linear sprint. The pooled results demonstrate that plyometric training is an effective method for enhancing lower-body explosive power in this athletic population, although the magnitude of effects and robustness of evidence varied across outcomes: moderate and statistically significant improvements were observed in both countermovement jump and squat jump height, confirming the training’s efficacy for vertical jump performance. Notably, the squat jump results exhibited minimal heterogeneity, suggesting a consistent training response, whereas considerable heterogeneity for countermovement jump indicated more variable responses influenced by individual or program differences. In contrast, the effect of plyometric training on the 20-meter sprint was smaller, less consistent, and non-significant, indicating a limited and uncertain transfer to linear sprint capability.

### Countermovement jump

Our analysis confirms that standalone plyometric training benefits countermovement jump (CMJ) performance in basketball players, rooted in stretch-shortening cycle (SSC)-mediated neuromuscular adaptations—including enhanced motor unit synchronization and elastic energy recoil—that augment lower-body explosive power (Komi, 2000; Suchomel et al., 2016). This improvement is clinically relevant for basketball, as CMJ ability directly underpins game-specific actions like rebounding, shot blocking, and vertical drives (Šarabon et al., 2020; Castagna et al., 2013), though considerable heterogeneity reflects variable responsiveness across populations. Age and sex emerge as key moderators: younger players exhibit more pronounced CMJ gains due to heightened neuromuscular plasticity during developmental maturation (Ramirez-Campillo et al., 2018; Malina, 2019), while adults show minimal improvements and may require complementary strength training to overcome performance plateaus (Bouteraa et al., 2020). Male athletes benefit significantly more than females, likely attributed to sex-related differences in muscle mass, fast-twitch fiber proportion, and SSC efficiency (Moran et al., 2019; Rae et al., 2021), compounded by a lack of female-specific training protocols tailored to physiological thresholds. Training duration does not drive superior outcomes—both shorter (≤6 weeks) and longer (>6 weeks) interventions yield meaningful improvements—indicating that program design (e.g., exercise type, intensity, specificity) is more influential (de Villarreal et al., 2009; Burgess et al., 2020). These findings support age- and sex-stratified training approaches, and future research should standardize interventions to address heterogeneity and expand evidence for underrepresented groups (Markovic and Mikulic, 2010; Suchomel et al., 2016).

### Squat jump

Based on the collective results from multiple studies, plyometric training consistently enhances squat jump performance in basketball players. Notably, (Khlifa et al., 2010a and Santos and Janeira, 2011), reported substantial improvements. The overall findings across all included studies demonstrate a clear positive effect of plyometric training on squat jump performance. This consistency in outcomes, observed despite variations in training protocols, suggests that plyometric training effectively improves concentric-dominant jump capacity regardless of potential moderating factors such as training duration or athlete level. These findings strongly support the efficacy of plyometric training for developing explosive lower-body power in basketball players, likely through specific neuromuscular adaptations that enhance SJ performance—a key indicator of concentric-dominant explosive strength associated with basketball-specific movements such as immediate vertical takeoff during jump shots or sudden rebound attempts.

### 20-meter sprint

The findings regarding the effect of plyometric training on 20-meter sprint performance present a conflicting picture relative to the existing literature. While some studies report meaningful improvements—such as Aksović et al. (2019), who observed notable enhancements in young basketball players—others demonstrate minimal effects, including investigations by de Villarreal et al. (2021) and Izquierdo Velasco (2022) that yielded negligible outcomes. Despite these divergent individual results, the collective findings indicate a consistent pattern of small but reliable improvements across the body of evidence. This suggests that while plyometric training does produce measurable benefits for sprint performance, these gains remain modest in magnitude. The limited transfer to linear sprint capability in basketball players likely stems from the distinct neuromuscular and technical demands of maximal velocity sprinting, which extend beyond the qualities developed through generic plyometric exercises (Afaq et al., 2024; Aksović et al., 2020; Komi, 2000; Suchomel et al., 2016). Plyometric training primarily targets stretch-shortening cycle efficiency for vertical explosive power, whereas sprint performance relies on optimized horizontal force application and speed-specific mechanics (Amato et al., 2018; Borenstein et al., 2010; Šarabon et al., 2020). Methodologically, the meta-analytic approach used to synthesize these effects adheres to established guidelines for heterogeneity assessment and effect size calculation (Higgins et al., 2003; IntHout et al., 2014; Lakens, 2013; Viechtbauer, 2010), ensuring the robustness of our findings. Consequently, plyometric training alone appears insufficient for substantially enhancing sprint performance, highlighting the need for more specific training modalities to optimize this athletic quality.

### Limitations and future research directions

Several limitations in this meta-analysis warrant consideration when interpreting the findings. First, a moderate risk of bias was identified across several domains, particularly in intervention classification, protocol deviations, and outcome measurement, largely attributable to non-standardized implementation and insufficient blinding. Second, substantial variability in intervention characteristics—including training duration, exercise selection, and session frequency—may have influenced the consistency and comparability of the results. Finally, although no statistically significant publication bias was detected for the 20-meter sprint outcomes, visual asymmetry in the countermovement jump funnel plot raises the possibility of an underrepresentation of null or negative results, which could lead to an overestimation of the true effect.

Priority should be given to conducting larger, high-quality randomized controlled trials with standardized intervention protocols and rigorous blinding procedures to minimize potential biases. In addition, researchers are encouraged to explore the dose-response relationship between plyometric training and performance outcomes by systematically varying training variables such as volume, intensity, and frequency. Future investigations should specifically examine the moderating effects of factors such as sex, age, training background, and competitive level to enhance our understanding of individualized training responses.

## Conclusion

This meta-analysis confirms that plyometric training effectively enhances vertical jump performance, especially squat jump, in basketball players, but provides minimal benefits for 20-m sprint performance. The considerable heterogeneity in countermovement jump outcomes indicates that training duration alone does not sufficiently explain efficacy variations, suggesting other programmatic factors are influential. Future studies should develop standardized interventions and further examine the role of athlete characteristics and training parameters to refine practice guidelines.

## Data Availability

The original contributions presented in the study are included in the article/Supplementary Material, further inquiries can be directed to the corresponding author.
