# Supplementary material for: Standalone plyometric training in basketball players: a meta-analytic comparison of countermovement jump, squat jump, and sprint performance enhancements
Source: Front Physiol. 2026 Mar 18;17:1747487. doi: 10.3389/fphys.2026.1747487 (PMC13038527; doi:10.3389/fphys.2026.1747487)
Supplement: Supplementary file 1 [file Table1.docx]

**Appendix 1：search strategy**

| **Database** | **Retrieval strategy** |
| --- | --- |
| **Cochranne** | **#1**(plyometric exercise OR plyometric* OR jump training OR explosive training OR stretch shortening cycle) |
|  | #2(basketball OR basketball player OR basketball players) |
|  | #3 #1 AND #2 |
| **Embase** | **#1**('plyometric exercise'/exp OR plyometric*:ab,ti OR 'jump training':ab,ti OR 'explosive training':ab,ti OR 'stretch shortening cycle':ab,ti)  #2 ('basketball'/exp OR basketball*:ab,ti OR 'basketball player*':ab,ti)  #3 #1 AND #2 |
| **Web of Science** | **#1**TS=("plyometric exercise" OR plyometric* OR "jump training" OR "explosive training" OR "stretch shortening cycle")  #2TS=("basketball" OR basketball* OR "basketball player*") |
|  | #3 #1 AND #2 |
| **PubMed** | **#1**(plyometric exercise OR plyometric* OR jump training OR explosive training OR stretch shortening cycle)  #2(basketball OR basketball player OR basketball players)  #3 #1 AND #2 |
| **Sportdiscus** | **#1**(AB(plyometric* OR "jump training" OR "explosive training" OR "stretch shortening cycle")  OR TI(plyometric* OR "jump training" OR "explosive training" OR "stretch shortening cycle")  OR DE "Plyometric Exercise") |
|  | #2(AB(basketball* OR "basketball player*")  OR TI(basketball* OR "basketball player*")  OR DE "Basketball") |
|  | #3 #1 AND #2 |
